# Frequent Constriction-Like Echocardiographic Findings in Elite Athletes Following Mild COVID-19: A Propensity Score-Matched Analysis

**DOI:** 10.3389/fcvm.2021.760651

**Published:** 2022-01-05

**Authors:** Bálint Károly Lakatos, Márton Tokodi, Alexandra Fábián, Zsuzsanna Ladányi, Hajnalka Vágó, Liliána Szabó, Nóra Sydó, Emese Csulak, Orsolya Kiss, Máté Babity, Anna Réka Kiss, Zsófia Gregor, Andrea Szűcs, Béla Merkely, Attila Kovács

**Affiliations:** ^1^Heart and Vascular Center, Semmelweis University, Budapest, Hungary; ^2^Department of Sports Medicine, Semmelweis University, Budapest, Hungary

**Keywords:** athlete's heart, COVID-19, speckle-tracking analysis, 3D echocardiography, constrictive pericaditis

## Abstract

**Background:** The cardiovascular effects of SARS-CoV-2 in elite athletes are still a matter of debate. Accordingly, we sought to perform a comprehensive echocardiographic characterization of post-COVID athletes by comparing them to a non-COVID athlete cohort.

**Methods:** 107 elite athletes with COVID-19 were prospectively enrolled (P-CA; 23 ± 6 years, 23% female) 107 healthy athletes were selected as a control group using propensity score matching (N-CA). All athletes underwent 2D and 3D echocardiography. Left (LV) and right ventricular (RV) end-diastolic volumes (EDVi) and ejection fractions (EF) were quantified. To characterize LV longitudinal deformation, 2D global longitudinal strain (GLS) and the ratio of free wall vs. septal longitudinal strain (FWLS/SLS) were also measured. To describe septal flattening (SF—frequently seen in P-CA), LV eccentricity index (EI) was calculated.

**Results:** P-CA and N-CA athletes had comparable LV and RVEDVi (P-CA vs. N-CA; 77 ± 12 vs. 78 ± 13mL/m2; 79 ± 16 vs. 80 ± 14mL/m2). P-CA had significantly higher LVEF (58 ± 4 vs. 56 ± 4%, *p* < 0.001), while LVGLS values did not differ between P-CA and N-CA (−19.0 ± 1.9 vs. −18.8 ± 2.2%). EI was significantly higher in P-CA (1.13 ± 0.16 vs. 1.01 ± 0.05, *p* < 0.001), which was attributable to a distinct subgroup of P-CA with a prominent SF (*n* = 35, 33%), further provoked by inspiration. In this subgroup, the EI was markedly higher compared to the rest of the P-CA (1.29 ± 0.15 vs. 1.04 ± 0.08, *p* < 0.001), LVEDVi was also significantly higher (80 ± 14 vs. 75 ± 11 mL/m2, *p* < 0.001), while RVEDVi did not differ (82 ± 16 vs. 78 ± 15mL/m2). Moreover, the FWLS/SLS ratio was significantly lower in the SF subgroup (91.7 ± 8.6 vs. 97.3 ± 8.2, *p* < 0.01). P-CA with SF experienced symptoms less frequently (1.4 ± 1.3 vs. 2.1 ± 1.5 symptom during the infection, *p* = 0.01).

**Conclusions:** Elite athletes following COVID-19 showed distinct morphological and functional cardiac changes compared to a propensity score-matched control athlete group. These results are mainly driven by a subgroup, which presented with some echocardiographic features characteristic of constrictive pericarditis.

## Introduction

The COVID-19 pandemic represents an unprecedented challenge to the healthcare systems worldwide with still increasing patient numbers. While the infection was initially thought to be affecting mainly the respiratory tract, current evidence suggests that the cardiovascular consequences of COVID-19 are not negligible (1). SARS-CoV-2-related myocardial injury is frequently reported as a worrisome manifestation, whereas prior cardiovascular disorders are strong negative prognostic factors for the course of the infection (2, 3).

Fortunately, COVID-19 is often asymptomatic or associated with only mild symptoms, especially in the young (4). Still, the potential cardiac effects of an uncomplicated SARS-CoV-2 infection need to be further explored.

Elite athletes are a distinguished group of young individuals as a relatively high proportion of them underwent (or will undergo) the infection. This is attributable to their high-risk profile: a young community with frequent social interactions; the majority of sport disciplines include direct physical contact; and wearing a mask during training sessions or competitions is rarely a realistic expectation (5). While the vast majority of young athletes experience an uncomplicated disease course, it is important to emphasize that high-intensity training and related cardiac adaptation may even exaggerate the adverse effects of COVID-19, as it does for other cardiac or non-cardiac disorders (6). Initial reports demonstrated that a considerable proportion of athletes may have detectable myocardial damage; however, the lack of proper control groups limited the generalizability of these results (7–10). Recent studies also proposed the possibility of pericardial involvement (10, 11). Nevertheless, all of the aforementioned studies utilized cardiac magnetic resonance (cMR), an imaging modality that can hardly be incorporated into the routine return to play examination protocol. As a potential alternative, the clinical value of state-of-the-art echocardiographic techniques, such as 3D echocardiography and speckle-tracking echocardiography (STE) should be also tested.

Accordingly, we sought to perform a comprehensive echocardiographic characterization of post-COVID athletes and compare them to a propensity score (PS)-matched healthy athlete cohort.

## Materials and Methods

### Patient Characteristics

We consecutively enrolled elite athletes undergoing “return to play” examinations between September and December 2020 at our Center's Sports Cardiology Department (study protocol approved by the National Public Health Center; no: ETT TUKEB IV/10282-1/2020/EKU). The study protocol complies with the Declaration of Helsinki, and participants gave written informed consent to every procedure. SARS-CoV-2 infection was diagnosed by real-time polymerase chain reaction (rt-PCR) or by serum immunoglobulin G (IgG) antibody titer measurement. All athletes were officially released from quarantine defined by having two negative rt-PCR assays of nasopharyngeal swab specimens following the infection and/or passing the appropriate quarantine period (10 or 14 days depending on the time of enrollment). All of the athletes completed a questionnaire regarding the nature and duration of their SARS-CoV-2 infection, based on the recommendation of the National Institute of Health (12). Detailed medical history and training regimen were obtained along with the routine physical examination and 12-lead electrocardiogram. Body surface area (BSA) was calculated using the Mosteller formula (13). Subjects with previously documented uncommon echocardiographic and/or electrocardiographic features or with suboptimal echocardiographic image quality for further analysis (*n* = 5) and athletes who suspended regular training in the preceding 6 months before their SARS-CoV-2 infection (*n* = 2) were excluded.

To enable the appropriate pairwise comparison of COVID vs. non-COVID athletes, PS-matching was performed with the optimal pair matching algorithm (14). Our institutional database comprising 425 elite athletes served as the pool for the matching. First, propensity scores were calculated based on age, BSA, and weekly training hours. Then, each COVID athlete was paired with one non-COVID athlete from our institutional database, targeting the collective optimization of the overall criterion (i.e., minimizing the mean of the within-pair difference in propensity score). Matching was applied in males and females separately to ensure that each COVID athlete is paired with a non-COVID athlete of the same sex. PS-matching was performed in R (version 3.6.3, R Foundation for Statistical Computing, Vienna, Austria) using the MatchIt package (version 3.0.2).

### Conventional Echocardiography

Echocardiographic loops were recorded using a Vivid E95 ultrasound system equipped with a 4Vc-D phased-array transducer (GE Vingmed Ultrasound, Horten, Norway). Cardiac chambers were quantified according to current guidelines (15). Left ventricular (LV) wall thicknesses and diameters were measured in the parasternal long-axis view at the level of mitral valve coaptation. Relative wall thickness (RWT) was calculated as 2^*^posterior wall thickness/LV end-diastolic internal diameter. LV diastolic eccentricity index was measured from parasternal short-axis view at the level of the papillary muscles, defined as the ratio of the distances between the anterior-to-posterior wall and the septal-to-lateral wall in end-diastole. Left- and right atrial volumes were measured using the Simpson method and were indexed to BSA. LV diastolic inflow by pulsed-wave Doppler at the level of the mitral valve coaptation was obtained to determine early (E) and late diastolic (A) peak velocities, their ratio, and E-wave deceleration time. Pulsed-wave tissue Doppler imaging (TDI) was used to measure systolic (s′), early (e′), and late diastolic (a′) velocities at the mitral lateral and medial annuli. The ratio of E-wave velocity to averaged e′ velocities of the mitral medial and lateral annuli was calculated, serving as an estimate of LV filling pressures. Tricuspid annular plane systolic excursion (TAPSE) was measured by M-mode as the peak longitudinal excursion of the tricuspid annulus on an apical four-chamber view. Inferior vena cava (IVC) diameters estimated right atrial pressure (RAP), pulmonary arterial systolic pressure (PASP), diastolic pressure (PADP), mean pressure (PAMP), and also pulmonary vascular resistance (PVR) were quantified according to the current echocardiographic recommendations (16). The presence of a visually detectable septal flattening or pericardial effusion was evaluated during postprocessing by a single expert operator (B.L.) blinded to the study groups.

### Speckle-Tracking Analysis

ECG-gated, LV-focused apical long axis, four- and two-chamber view loops targeting a frame rate over 50 FPS were obtained for further analysis. STE was performed by a single expert operator (B.L.) blinded to the study groups using dedicated semi-automatic software (EchoPAC v204 AFI, GE). The software automatically detects the myocardial region of interest (ROI) of the given acquisition and tracks its motion throughout the cardiac cycle. If necessary, the ROI was adjusted manually in order to provide adequate tracking. Segments with poor tracking quality (driven by the software's recommendation) were excluded from the analysis; however, subjects with three or more excluded segments were not included in the study (none). The software automatically calculates global longitudinal strain (GLS) and segmental longitudinal strain (LS) values as well. By averaging the segmental data of the free wall (FW—average LS of inferior, posterior, lateral, and anterior segments) and septal (S—average LS of infero- and anteroseptal segments) regions, we have quantified FWLS and SLS, respectively.

### 3D Echocardiography

LV- and RV-focused ECG-gated full volume 3D datasets were obtained from apical four-chamber view using multi-beat reconstruction from 4 cardiac cycles. Offline analyses of these datasets focused on the LV and RV were performed by the same expert, blinded operator using conventionally available software packages (4D LV Analysis 3 and RV-Function 2, TomTec Imaging Systems GmbH, Unterschleissheim, Germany). The algorithm automatically generates LV and RV endocardial contours, which were manually corrected on multiple short- and long-axis planes throughout the entire cardiac cycle. We determined the LV and RV end-diastolic volume index (EDVi), end-systolic volume index (ESVi), and stroke volume index (SVi) normalized to BSA. To quantify global ventricular function, LV and RV ejection fractions (EF) were also calculated.

### Statistical Analysis

All values are expressed as mean ± standard deviation, or median and interquartile range (IQR). The distribution of the variables was assessed by the Shapiro-Wilk normality test. An unpaired two-sided Student's *t*-test, in case of normal distribution, or a Mann-Whitney U test, in case of non-normal distribution, was performed to compare the continuous variables of the study groups. Fisher's exact test was used to compare the incidence of symptoms between groups. A *p* < 0.05 was used as the criterion for statistical significance.

Intra- and interobserver variability of the most relevant parameters were also assessed. The operator of the first measurements (B.L.) and a second expert reader (A.F.), both blinded to the study groups, repeated the measurements in a randomly chosen subset of 5–5 athletes from each group. Lin's concordance correlation coefficient and coefficient of variation were calculated.

## Results

One hundred and seven post-COVID athletes (handball *n* = 37, ice hockey *n* = 26, water polo *n* = 26, basketball *n* = 12, speedskating *n* = 2, other *n* = 4) were included in the current analysis. Athletes were asymptomatic at the time of examination with the exception of the loss of taste and/or smell in a handful of cases (*n* = 12), as these symptoms frequently exceed the period of active infection (17). A total of 59 subjects (55%) were completely asymptomatic throughout the disease course. The symptom burden of the study group is summarized in Supplementary Table 1. The athletes were symptomatic for a median of 4 [IQR: 1–7] days and presented for the return to play examinations 22 [IQR: 17–25] days following the first rt-PCR or IgG positivity.

The mean age of the post-COVID athletes was 23 ± 6 years. There were no differences in age, BSA and training hours between the post-COVID and the PS-matched non-COVID athletes, indicating successful matching. Systolic and diastolic blood pressures also did not differ between post-COVID and non-COVID athletes, while heart rate was significantly lower in the post-COVID group (Table 1).

**Table 1:** Baseline characteristics of the post-COVID and the non-COVID athlete groups.

	**Post-COVID athletes (*n =* 107)**	**Non-COVID athletes (*n =* 107)**	***p*-value**
Age (years)	22.9 ± 6.1	22.7 ± 7.0	0.82
Female (n [%])	25 (23%)	25 (23%)	1
Height (cm)	182.9 ± 10.0	181.8 ± 12.0	0.45
Weight (kg)	80.2 ± 15.3	80.6 ± 17.0	0.87
BSA (m^2^)	2.0 ± 0.2	2.0 ± 0.3	0.93
SBP (mmHg)	130.3 ± 15.1	134.0 ± 15.8	0.09
DBP (mmHg)	79.4 ± 11.3	77.4 ± 9.2	0.16
HR (1/min)	62.9 ± 10.6	66.6 ± 13.3	**<0.05**
Training per week (hours)	13.1 ± 6.0	14.5 ± 6.4	0.08

*BSA, body surface area; SBP, systolic blood pressure; DBP, diastolic blood pressure. HR, heart rate. Bold values indicate a p <0.05*.

Basic echocardiographic parameters of the left and right heart are shown in Table 2. LV wall thicknesses and RWT were significantly lower in the post-COVID group. Transmitral E/A ratio was higher in the post-COVID group, along with a longer deceleration time. TDI-derived mitral lateral and medial velocities were significantly higher in the post-COVID group resulting in a lower E/e′ ratio; however, e′ lateral/e′ medial ratio was significantly lower. 2D RV, left and right atrial dimensions did not differ between the study groups. Maximal IVC diameter and right atrial pressure were significantly lower in the post-COVID group, whereas other estimated pulmonary artery pressures were comparable between post-COVID athletes and PS-matched non-COVID athletes. TAPSE/PASP ratio was also similar (Table 2).

**Table 2:** Conventional echocardiographic left- and right heart parameters in the post-COVID and the non-COVID athlete groups.

	**Post-COVID athletes (*n =* 107)**	**Non-COVID athletes (*n =* 107)**	***p*-value**
LVIDd (mm)	51.8 ± 4.4	51.4 ± 5.4	0.56
IVSd (mm)	9.4 ± 1.8	10.4 ± 1.8	**<0.01**
PWd (mm)	8.4 ± 1.3	9.0 ± 1.3	**<0.01**
RWT (%)	0.33 ± 0.05	0.35 ± 0.05	**<0.001**
LAVi (mL/m^2^)	26.4 ± 6.5	27.9 ± 8.6	0.16
Transmitral E wave (cm/s)	81.7 ± 16.0	82.3 ± 20.6	0.79
Transmitral A wave (cm/s)	50.2 ± 12.3	57.4 ± 15.5	**<0.001**
E/A	1.68 ± 0.40	1.49 ± 0.43	**<0.001**
DT (ms)	192.7 ± 40.8	176.6 ± 39.3	**<0.01**
E/e′ average	4.64 ± 0.88	5.55 ± 1.50	**<0.001**
Mitral lateral s′ (cm/s)	12.8 ± 2.5	12.1 ± 2.3	**<0.05**
Mitral lateral e′ (cm/s)	19.7 ± 3.2	17.7 ± 3.2	**<0.001**
Mitral lateral a′ (cm/s)	8.3 ± 2.0	7.6 ± 1.8	**<0.01**
Mitral medial s′ (cm/s)	10.3 ± 1.5	9.6 ± 1.4	**<0.01**
Mitral medial e′ (cm/s)	15.6 ± 2.7	13.0 ± 2.6	**<0.001**
Mitral medial a′ (cm/s)	8.4 ± 1.4	7.5 ± 1.8	**<0.001**
e′ lateral/e′ septal	1.29 ± 0.21	1.40 ± 0.27	**<0.001**
LV diastolic eccentricity index	1.13 ± 0.16	1.01 ± 0.05	**<0.001**
RV basal diameter (mm)	34.3 ± 4.2	33.7 ± 4.3	0.27
TAPSE (mm)	24.7 ± 3.9	23.6 ± 4.2	0.05
RAVi (mL/m^2^)	28.0 ± 6.6	28.1 ± 8.1	0.89
PASP (mmHg)	20.7 ± 4.3	20.4 ± 5.2	0.61
PADP (mmHg)	6.9 ± 2.3	7.0 ± 2.8	0.76
PAMP (mmHg)	13.4 ± 4.2	12.3 ± 3.7	0.19
IVC max (mm)	13.2 ± 3.0	16.0 ± 4.1	**<0.001**
IVC min (mm)	11.3 ± 6.0	9.3 ± 6.7	0.39
RAP (mmHg)	3.5 ± 1.8	4.2 ± 2.3	**<0.05**
RVOT VTI (cm)	20.0 ± 3.5	18.8 ± 3.4	**<0.05**
PVR (Wood units)	1.24 ± 0.21	1.21 ± 0.26	0.51
TAPSE/PASP	1.23 ± 0.30	1.24 ± 0.42	0.86
Prevalence of mild pericardial effusion (n [%])	41 (38%)	10 (9%)	**<0.001**

*LVIDd, left ventricular end-diastolic diameter; IVSd, interventricular septal thickness; PWd, posterior wall thickness; RWT, relative wall thickness; LAVi, left atrial volume index; DT: deceleration time; LV eccentricity index, left ventricular eccentricity index; RV basal diamater, right ventricular basal diameter; TAPSE, tricuspid annular plane systolic excursion; RAVi, right atrial volume index; PASP, pulmonary arterial systolic pressure; PADP, pulmonary arterial diastolic pressure; PAMP, pulmonary arterial mean pressure; IVC, inferior vena cava; RAP, right atrial pressure; RVOT VTI, right ventricular outflow tract velocity-time integral; PVR, pulmonary vascular resistance. Bold values indicate a p <0.05*.

3D echocardiographic and 2D LV STE parameters are summarized in Table 3. 3D LV and RV EDVi were comparable between groups, whereas 3D LV ESVi was significantly lower in post-COVID athletes, resulting in elevated LV EF. 2D GLS, SLS, and FWLS were comparable between the post-COVID and non-COVID groups; however, a lower FWLS/SLS ratio was detected in the post-COVID athletes.

**Table 3:** Comparison of 3D and speckle-tracking echocardiographic data between the post-COVID and the non-COVID athlete groups.

	**Post-COVID athletes (*n =* 107)**	**Non-COVID athletes (*n =* 107)**	***p*-value**
3D LVEDVi (mL/m^2^)	76.7 ± 12.2	78.3 ± 13.3	0.39
3D LVESVi (mL/m^2^)	32.4 ± 6.3	34.7 ± 7.4	**0.01**
3D LVSVi (mL/m^2^)	44.4 ± 7.5	43.5 ± 7.3	0.4
3D LVEF (%)	57.9 ± 4.3	55.8 ± 4.2	**<0.001**
3D RVEDVi (mL/m^2^)	78.9 ± 15.5	79.6 ± 14.2	0.72
3D RVESVi (mL/m^2^)	35.4 ± 8.4	36.6 ± 8.6	0.32
3D RVSVi (mL/m^2^)	43.5 ± 8.5	43.1 ± 7.1	0.72
3D RVEF (%)	55.3 ± 4.5	54.3 ± 4.7	0.14
2D LVGLS (%)	−19.0 ± 1.9	−18.8 ± 2.2	0.51
2D FWLS (%)	−18.6 ± 2.1	−18.6 ± 2.2	0.97
2D SLS (%)	−19.6 ± 2.1	−19.0 ± 2.4	0.06
2D FWLS/SLS (%)	95.5 ± 8.7	98.3 ± 6.8	**<0.01**

*LVEDVi, left ventricular end-diastolic index; LVESVi, left ventricular end-systolic volume index; LVSVi, left ventricular stroke volume index; LVEF, left ventricular ejection fraction; RVEDVi, right ventricular end-diastolic volume index; RVESVi, right ventricular end-systolic volume index; RVSVi, right ventricular stroke volume index; RVEF, right ventricular ejection fraction; LVGLS, left ventricular global longitudinal strain; FWLS, free wall longitudinal strain; SLS, septal longitudinal strain; FWLS/SLS, free wall to septal longitudinal strain ratio. Bold values indicate a p <0.05*.

**Figure 1:**
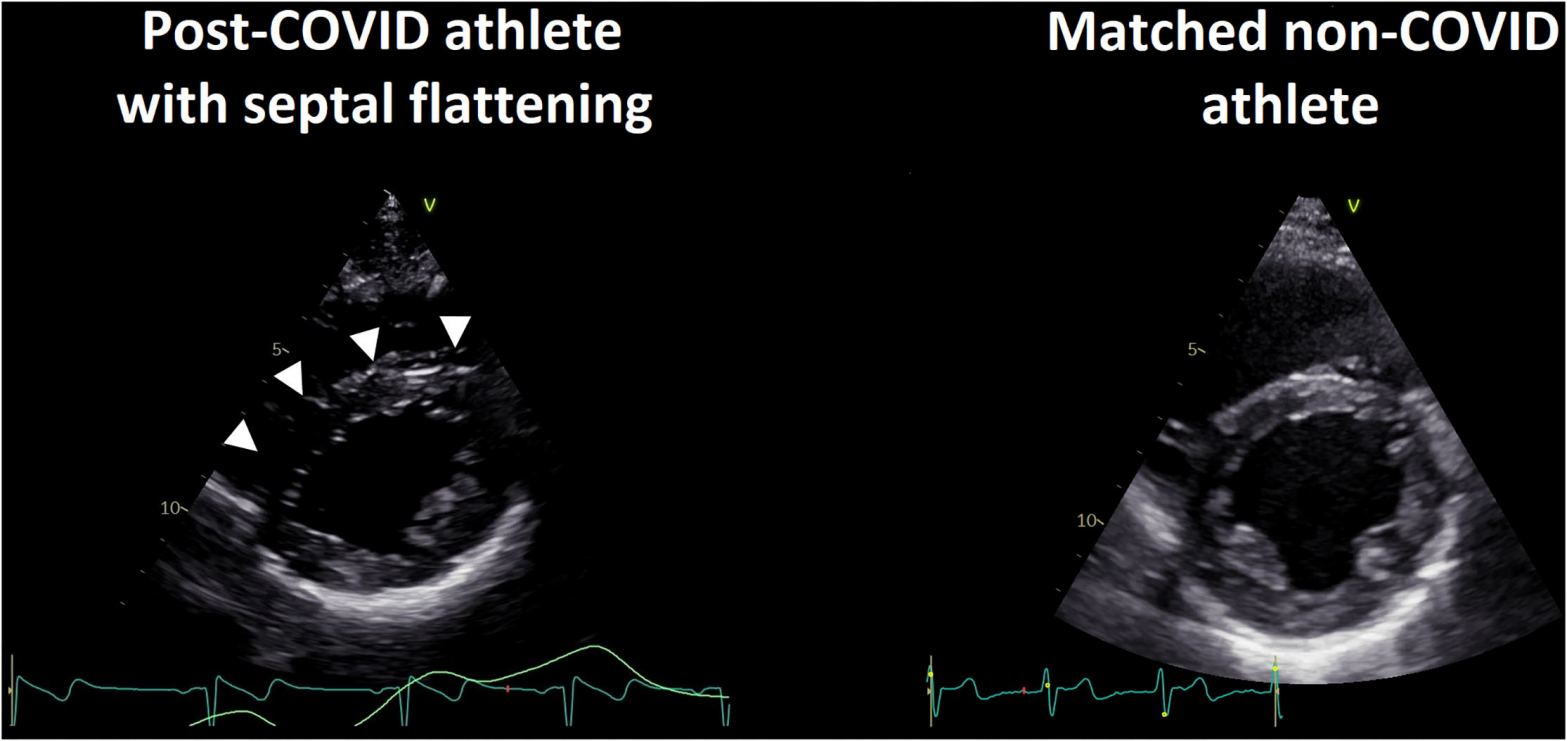
Representative case of the post-COVID septal flattening (SF) in athletes. Parasternal short-axis views at the level of the papillary muscles at mid-diastole in a young athlete underwent asymptomatic SARS-CoV-2 infection and his matched control. In the post-COVID athlete, a prominent SF can be seen with early diastolic dominance and inspiratory enhancement (left, SF shown by arrows), compared to the propensity score-matched control (right).

Interestingly, LV diastolic eccentricity index was significantly higher in the post-COVID subjects (1.13 ± 0.16 vs. 1.01 ± 0.05, *p* < 0.001). This finding was mainly driven by a subgroup (*n* = 35/107; 33%) of post-COVID athletes, in which an early-diastolic septal flattening (SF) was present consistently throughout the entire echocardiographic examination on multiple views, showing an inspiratory enhancement (Figure 1, Supplementary Video 1). This phenomenon was not detected in any athletes of the PS-matched non-COVID group. Therefore, we have also assessed the differences between the athletes with and without SF within the post-COVID group.

Post-COVID athletes with SF were younger; however, they did not differ in other anthropometric or basic hemodynamic measures and in average weekly training hours (Supplementary Table 2).

**Figure 2:**
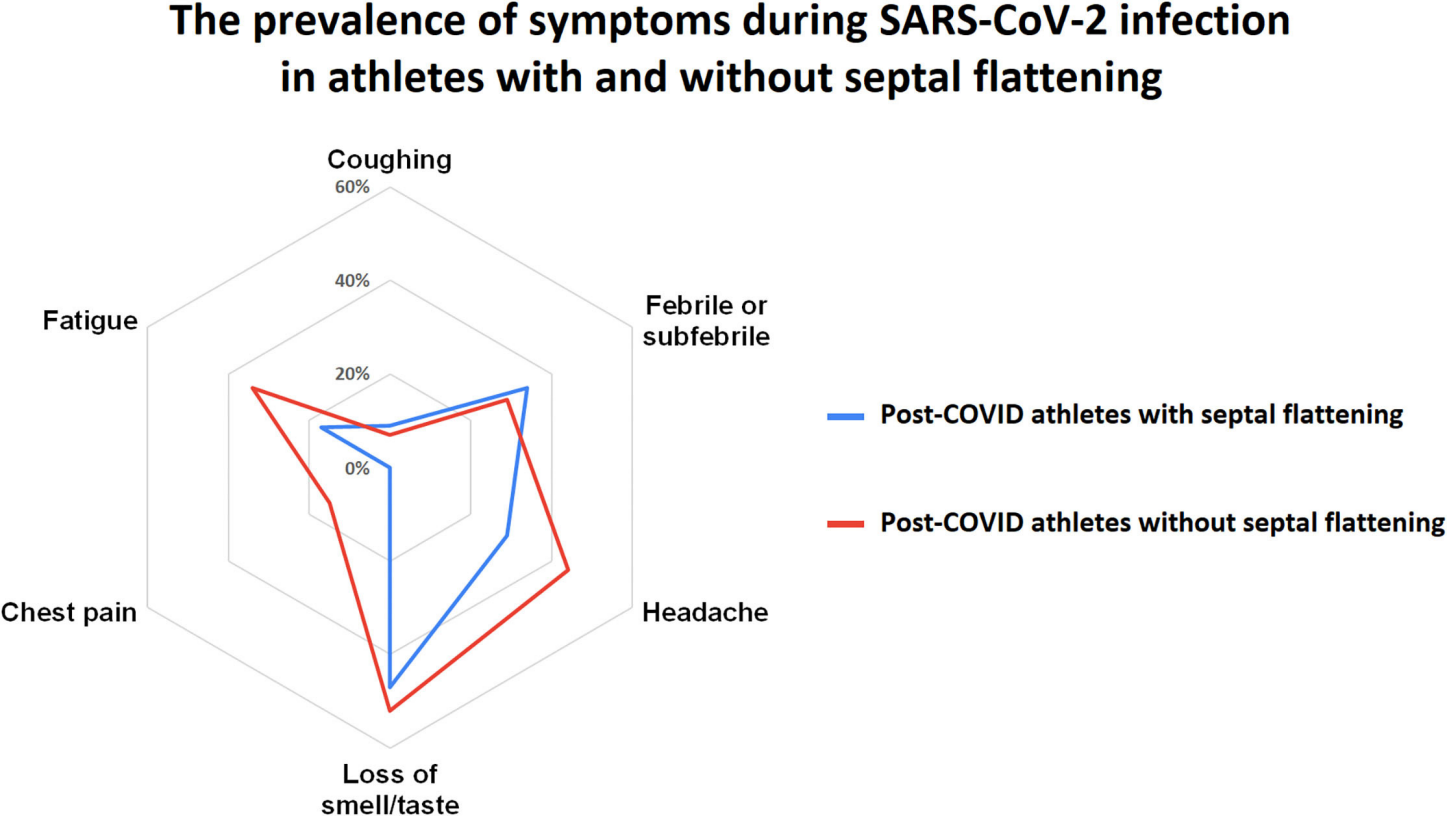
Radar chart comparisons of the most relevant symptoms in post-COVID athletes with our without septal flattening (SF). Athletes with SF (blue line) and without SF (red line) did not differ in the incidence of fever or subfebrility, coughing, headache or the lost of smell and/or taste. On the other hand, chest pain and fatigue were significantly more frequent in athletes without SF. In general, athletes with SF were less symptomatic, as shown by the smaller area of the radar chart compared to athletes without SF (see details in text).

The presence of detectable (trivial) pericardial effusion was more frequent in the SF subgroup of post-COVID athletes compared to the corresponding subset of PS-matched non-COVID athletes (41% vs. 12%, *p* < 0.01). Post-COVID athletes with SF and without SF did not differ in terms of the number of symptomatic days (3 [IQR: 0–7.0] days vs. 5 [IQR: 2.5–8.0] days, *p* = 0.09), the time between the onset of symptoms and the examination (24 [IQR: 17.5–37.5] days vs. 23 [IQR: 18.0–29.0] days, *p* = 0.65), or the time elapsed between the first positive PCR or IgG and the examination (22.5 [IQR: 17.0–25.0] days vs. 21 [IQR: 17.0–25.0] days, *p* = 0.70). The incidence of fever (34 vs. 29%, *p* = 0.66), coughing (9 vs. 7%, *p* = 0.71), headache (29 vs. 44%, *p* = 0.15), and loss of smell and/or taste (47 vs. 52%, *p* = 0.54) were also comparable between the athlete groups. Interestingly, chest pain (0 vs. 15%, *p* = 0.01) and fatigue (17 vs. 34%, *p* = 0.04) were reported more frequently in post-COVID athletes without SF (Figure 2). When the symptom burden was summed as a “composite symptom score”, athletes with SF generally had fewer symptoms (1.4 ± 1.3 vs. 2.1 ± 1.5 symptom during the infection, *p* = 0.01, Figure 2).

**Figure 3:**
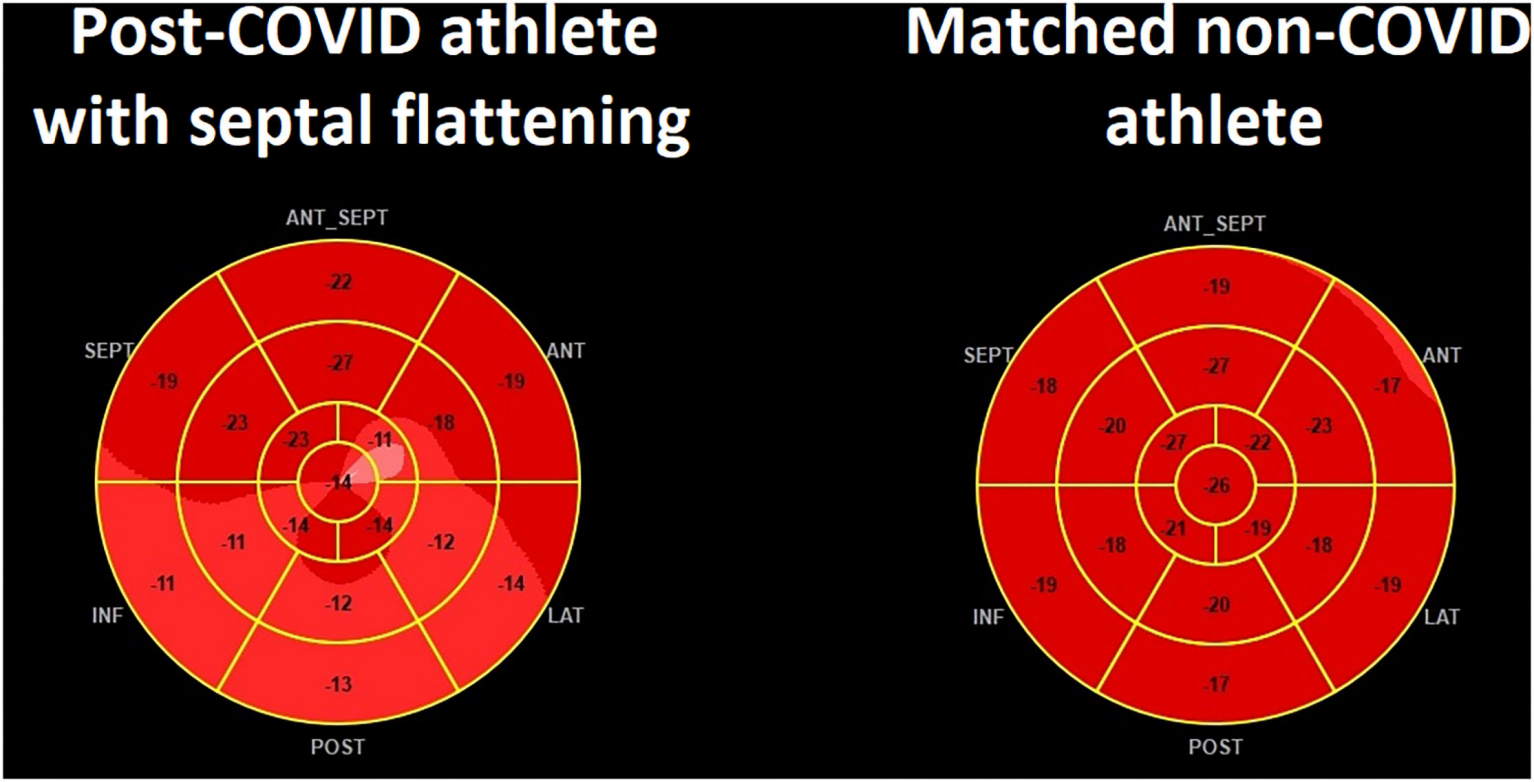
The “Hot Septum Sign” in a post-COVID athlete with septal flattening. While left ventricular global longitudinal strain is preserved, a relative decrease in the free wall segments can be noted (left), suggestive of a characteristic feature of pericardial constriction. In the matched control, segmental strain values of the septum and free wall do not markedly differ (right).

Regarding basic echocardiographic measures, post-COVID athletes with SF showed significantly higher E/A ratio, while RAP and PADP were also found to be significantly higher compared to post-COVID athletes without SF (Supplementary Table 2). LV diastolic eccentricity index was markedly higher in post-COVID athletes with SF, while it was comparable between post-COVID athletes without SF and their matched non-COVID athletes (1.04 ± 0.08 vs. 1.00 ± 0.04, *p* = 0.14). Regarding 3D echocardiographic measures, post-COVID athletes with SF had significantly higher LV EDVi and LV ESVi compared to post-COVID athletes without SF, while RV morphological measures along with LV and RV EF were similar (Table 4). 2D LV GLS did not differ between the post-COVID athlete subgroups; however, the FWLS/SLS ratio was significantly lower in athletes with SF compared to those without (Figure 3). During the last phase of the enrollment and already having our awareness at SF and related STE-based alterations, we have referred athletes presented with SF to cMR examination (*n* = 5). Notably, no myopericardial involvement was detected by cMR in these cases. Detailed case reports are presented in Supplementary Table 3.

**Table 4:** Echocardiographic comparison of post-COVID athletes with vs. without septal flattening.

	**Post-COVID athletes with SF (*n =* 35)**	**Post-COVID athletes without SF (*n =* 72)**	***p*-value**
LV diastolic eccentricity index	1.29 ± 0.15	1.04 ± 0.08	**<0.001**
3D LVEDVi (mL/m^2^)	80.1 ± 14.4	74.9 ± 10.7	**<0.05**
3D LVESVi (mL/m^2^)	34.5 ± 8.0	31.3 ± 5.1	**<0.05**
3D LVSVi (mL/m^2^)	46.2 ± 8.2	43.5 ± 7.1	0.09
3D LVEF (%)	57.5 ± 4.6	58.1 ± 4.1	0.52
3D RVEDVi (mL/m^2^)	82.1 ± 15.9	77.7 ± 15.3	0.3
3D RVESVi (mL/m^2^)	36.5 ± 9.8	35.9 ± 7.6	0.37
3D RVSVi (mL/m^2^)	44.7 ± 7.8	42.9 ± 8.8	0.31
3D RVEF (%)	55.6 ± 5.5	55.2 ± 4.0	0.68
2D LVGLS (%)	−18.9 ± 1.9	−19.0 ± 2.0	0.70
2D FWLS (%)	−18.3 ± 2.0	−18.8 ± 2.1	0.20
2D SLS (%)	−20.0 ± 2.3	−19.4 ± 2.0	0.16
2D FWLS/SLS (%)	91.7 ± 8.6	97.3 ± 8.2	**<0.001**

*SF, septal flattening; LVEDVi, left ventricular end-diastolic index; LVESVi, left ventricular end-systolic volume index; LVSVi, left ventricular stroke volume index; LVEF, left ventricular ejection fraction; RVEDVi, right ventricular end-diastolic volume index; RVESVi, right ventricular end-systolic volume index; RVSVi, right ventricular stroke volume index; RVEF, right ventricular ejection fraction; LVGLS, left ventricular global longitudinal strain; FWLS, free wall longitudinal strain; SLS, septal longitudinal strain; FWLS/SLS, free wall to septal longitudinal strain ratio. Bold values indicate a p <0.05*.

Intra- and interobserver variability of the key echocardiographic parameters showed good intra- and interreader agreements (Supplementary Table 4).

## Discussion

Our study is the first to investigate a relatively high number of European elite athletes who underwent mild COVID-19, while also comparing them to a PS-matched healthy athlete group using a comprehensive echocardiographic approach. We have shown that post-COVID athletes show distinct changes in cardiac morphology and function compared to matched non-COVID athletes. Of note, the vast majority of these alterations was attributable to a subpopulation of athletes in whom an inspiration-enhanced early diastolic SF could be detected. In these athletes, the E/A ratio of mitral inflow, the 3D echocardiography-derived LV volumes were significantly higher, along with a significantly lower FWLS/SLS ratio.

The earliest reports from China already mentioned the high prevalence of elevated cardiac necroenzymes and the commonly deteriorated LV functional measures in COVID-19 patients (18). With the worldwide expansion of the pandemic, several other studies demonstrated the high frequency of cardiac damage; however, the investigations were mainly focused on the severe/critical cases (19). Nowadays, evidence is growing that mild or even asymptomatic disease courses do not exclude myocardial involvement of COVID-19 (20). Special considerations are needed in the case of elite athletes following SARS-CoV-2 infection, even though these young, exceptionally healthy individuals usually undergo COVID-19 with no or very mild symptoms. Robust evidence suggests that even minor cardiac alterations can be exaggerated by high-intensity exercise, and this can worsen the course of various diseases (6). Therefore, the detailed characterization of the athlete's heart following COVID-19 is a relevant clinical demand.

In our post-COVID population, LV wall thicknesses and RWT were significantly lower compared to the matched control athletes. These findings may correspond to the effects of short-term detraining: LV wall thicknesses are known to decrease even after a few weeks of suspended athletic activity along with unaltered ventricular volumes (21, 22). Regarding functional measures, LV EF was found to be significantly higher in the post-COVID group, with unaltered LV GLS. While data are conflicting regarding the resting LV systolic function of the athlete's heart, low-normal values are commonly reported; therefore, an increase in LV EF following detraining may be expected (23).

In a subpopulation of our post-COVID elite athletes, an early diastolic SF was detected with inspiratory enhancement, commonly resulting in marked LV eccentricity (Figure 1, Supplementary Video 1). This phenomenon may be attributable to a handful of causes. Previous studies examining sedentary COVID-19 patients and also elite athletes reported alterations of the myocardium with a septal predominance (such as decreased septal LS, or increased native T1 values and/or late gadolinium enhancement of the septum) suggestive of SARS-CoV-2-related myocarditis (7, 11). Nevertheless, in viral myocarditis, segmental or global wall motion abnormalities would be expected rather than a bouncing septal motion with preserved deformation.

COVID-19 is also known to affect the pulmonary vasculature (24). However, in our post-COVID cohort, Doppler-based estimated pulmonary pressures did not markedly differ from matched control athletes, and post-COVID athletes with SF were also comparable to those without SF regarding these measures. Of note, estimated RAP and PADP were significantly higher in athletes with SF, nevertheless, only with a borderline statistical significance. Moreover, a COVID-related imbalance between intrapericardial and intrathoracic pressures should also be considered.

The aforementioned study of Brito et al. reported a surprisingly high prevalence of pericardial involvement in their study enrolling student athletes (11). While acute viral pericarditis does not usually alter myocardial function, previous results suggest that a transient pericardial constriction-like physiology may occur, which could explain the SF (25). As a marker of a possible pericardial inflammation, the prevalence of a detectable (although trivial) pericardial effusion was significantly higher in our post-COVID athletes compared to their matched non-COVID athletes. Furthermore, this constriction-like behavior is also reinforced by the phenomenon that the SF seems to be enhanced by inspiration and becomes the most prominent during early diastole (Figure 1) (26). Regarding STE-markers, in our post-COVID athletes with SF, the characteristic “hot septum” sign can be seen as shown by the STE-derived FWLS/SLS ratio (Figure 3) (27, 28). Of note, it is important to mention that other, less specific markers of constrictive physiology, such as increased E/A ratio and PADP, can also be measured in this subpopulation. In line with the cMR findings of the post-COVID population of Brito and colleagues, LV volumes were significantly higher in the post-COVID group with SF (11). This may be attributable to the partially similar methodology of cMR and a “multi-beat” 3D echocardiographic acquisition: during expiratory breathhold, the enhanced ventricular interdependence of the constrictive physiology may result in increased LV volumes (26).

Interestingly, SF was more common in athletes with generally fewer symptoms. This corresponds to previous large-scale cMR data demonstrating that all athletes with confirmed inflammatory heart disease were only minimally symptomatic (9). Moreover, in another study, pericardial enhancement was significantly more common in asymptomatic athletes (11). In a large retrospective cardiac surgery registry, post-surgery constrictive pericarditis patients were characterized by more commonly detected postoperative pericardial effusion and a higher LVEF (29). These results may indicate that the main driver of disease progression is the interplay of ongoing serous membrane inflammation and more pronounced friction of the pericardial sac by a hyperdynamic LV. Theoretically, athletes with fewer symptoms are likely to continue training during the infection, potentially creating a similar pathophysiological scenario.

However, it is important to mention that in those cases where cMR was also performed, no signs of pericardial inflammation or constriction were detected. Considering that the main body of data about the reverse remodeling after abrupted training is derived from small sample studies of the early 90's, it is plausible that a temporary change in the pericardial constraint is a benign phenomenon of athletic detraining (HIV). Hemodynamic overload is proved to induce not only myocardial, but pericardial remodeling as well (HIV). Therefore, it is suspected that intense regular exercise may also induce changes of the pericardial structure. While myocardial deconditioning is known to take place over the course of a few weeks of abrupted training regime, the altered characteristics of the pericardium may persist for a longer time, resulting in temporary changes of the pericardial constraint.

The current Sports Cardiology and Exercise Guideline of the European Society of Cardiology recommends at least 30 days of suspended training in the case of pericarditis, however, in the absence of a proven inflammatory process the clinical implications of these findings are hard to judge (HIV).In the case of persistent constriction-like changes in the SF post-COVID group, an impaired peak exercise capacity has high possibility (HIV)., Follow-up of athletes and further research are urged to explain the appearance and the potential clinical consequences of the constriction-like echocardiographic findings in the context of the athlete's heart.

## Limitations

Our study carries limitations that have to be acknowledged for adequate interpretation. First, our case number is limited. Nevertheless, the number of subjects is considered to be relatively high as compared to current COVID-19-related data in athletes. The population has a male predominance; therefore, the study was not powered to examine the role of gender differences. In the post-COVID athlete group, echocardiographic loops prior to the SARS-CoV-2 infection were not available. Therefore, PS-matching was used to provide a matched control athlete group. The observed changes were often subtle and only statistically significant. The most common causes of SF are pulmonary hypertension and constrictive pericarditis, and the gold-standard evaluation method for both these diseases is still right (and left-) heart catheterization (26). For obvious ethical reasons, such invasive procedures were not performed in the mainly asymptomatic/paucisymptomatic post-COVID athlete group. However, various echocardiographic pressure estimates and other functional parameters were quantified, which may also adequately assess the characteristic features of such diseases. Nevertheless, certain indirect markers of pericardial constriction, such as mitral and/or tricuspid inflow variation, hepatic vein flow and M-mode assessment of the septal motion were not obtained. Computed tomography was not included in this study; therefore, possible pulmonary involvement of the post-COVID athletes was not evaluated. cMR examinations were only performed in a handful of athletes; therefore, the gold-standard measurements of cardiac volumes are not available, and cMR markers of myopericardial inflammation were not assessed in the majority of the subjects. The cMR acquisitions were obtained during breath holding, therefore, free breathing loops confirming the septal flattening were not available. Respirometry was not part of our routine echocardiographic image acquisition protocol (used only in a few cases in the post-COVID group); therefore, the inspiratory enhancement of the SF was not tested consistently. Assessment of the long-term consequences and clinical importance of these findings requires further work and follow-up.

## Conclusions

Our results suggest that even mild SARS-CoV-2 infection may significantly affect cardiac morphology and function in elite athletes. The observed alterations are mainly attributable to a subgroup of athletes, in whom some features of pericardial constriction could be detected, such as pericardial effusion, early diastolic SF with inspiratory enhancement, and STE-derived “hot septum” sign. Interestingly, these athletes seemed to experience fewer symptoms during the course of the infection. Considering that current guidelines usually propose a more thorough return to play examinations in symptomatic athletes only, our data is especially alarming, as many of our athletes presented with SF would not have been eligible for a detailed assessment (30). The pathophysiological background and clinical relevance of these findings are unclear and require further research. Nevertheless, our data support the use of a comprehensive echocardiographic protocol applying advanced techniques in the return to play examination of elite athletes.

## Data Availability Statement

The original contributions presented in the study are included in the article/Supplementary Materials, further inquiries can be directed to the corresponding author/s.

## Ethics Statement

The studies involving human participants were reviewed and approved by National Public Health Center; No: ETT TUKEB IV/10282-1/2020/EKU. Written informed consent to participate in this study was provided by the participants' legal guardian/next of kin.

## Author Contributions

BL, MT, AF, MB, and AK contributed to the conception of the study design. BL, HV, LS, NS, EC, OK, MB, AK, ZG, and AS performed the measurents. BL, MT, AF, ZL, LS, and EC managed the database. BL and MT perfomed the statistical analysis. BL, MB and AK wrote the draft of the manuscript. MT, AF, HV, LS, NS, EC, OK, AS, and MB reviewed it. BL, MT, and MB prepared the figures. All authors contributed to the article and approved the submitted version.

## Funding

Project no. NVKP_16-1-2016-0017 (National Heart Program) has been implemented with the support provided from the National Research, Development and Innovation Fund of Hungary, financed under the NVKP_16 funding scheme. This research was financed by the Thematic Excellence Programme (2020-4.1.1.-TKP2020) of the Ministry for Innovation and Technology in Hungary, within the framework of the Therapeutic Development and Bioimaging Thematic Programmes of the Semmelweis University and also by the New National Excellence Program of the Ministry of Human Capacities (ÚNKP-20-4-I-SE-13 to BL, ÚNKP-20-3-I-SE-41 to MB, and ÚNKP-20-5 to AK). This project was also supported by a grant from the National Research, Development and Innovation Office (NKFIH) of Hungary (K135076 to BM). AK was supported by the János Bolyai Research Scholarship of the Hungarian Academy of Sciences. This project was supported by a grant from the National Research, Development and Innovation Office (NKFIH) of Hungary (2020-1.1.6-JÖVŐ-2021-00013).

## Conflict of Interest

The authors declare that the research was conducted in the absence of any commercial or financial relationships that could be construed as a potential conflict of interest.

## Publisher's Note

All claims expressed in this article are solely those of the authors and do not necessarily represent those of their affiliated organizations, or those of the publisher, the editors and the reviewers. Any product that may be evaluated in this article, or claim that may be made by its manufacturer, is not guaranteed or endorsed by the publisher.

## Supplementary Material

Supplementary Video 1 Post-COVID athlete with septal flattening (SF). An early diastolic dominance of the SF can be seen with inspiratory enhancement (as shown on the parallel ECG and respirometry tracing).

## References

[1] Guzik TJ Mohiddin SA Dimarco A Patel V Savvatis K Marelli-Berg FM. COVID-19 and the cardiovascular system: implications for risk assessment, diagnosis, and treatment options. Cardiovasc Res. (2020) 116:1666–87. 10.1093/cvr/cvaa106 32352535 PMC7197627

[2] Peng Y Meng K He M Zhu R Guan H Ke Z. Clinical characteristics and prognosis of 244 cardiovascular patients suffering from coronavirus disease in Wuhan, China. J Am Heart Assoc. (2020) 9:e016796. 10.1161/JAHA.120.016796 32794415 PMC7792394

[3] Bae S Kim SR Kim MN Shim WJ Park SM. Impact of cardiovascular disease and risk factors on fatal outcomes in patients with COVID-19 according to age: a systematic review and meta-analysis. Heart. (2021) 107:373–80. 10.1136/heartjnl-2020-317901 33334865

[4] Barek MA Aziz MA Islam MS. Impact of age, sex, comorbidities and clinical symptoms on the severity of COVID-19 cases: a meta-analysis with 55 studies and 10014 cases. Heliyon. (2020) 6:e05684. 10.1016/j.heliyon.2020.e05684 33344791 PMC7737518

[5] Watson A Haraldsdottir K Biese K Goodavish L Stevens B McGuine T. The association of COVID-19 incidence with sport and face mask use in United States high school athletes. medRxiv. (2001) 2019.21250116. 10.4085/1062-6050-281-21 34793596 PMC9913054

[6] Guasch E Mont L. Diagnosis, pathophysiology, and management of exercise-induced arrhythmias. Nat Rev Cardiol. (2017) 14:88–101. 10.1038/nrcardio.2016.173 27830772

[7] Rajpal S Tong MS Borchers J Zareba KM Obarski TP Simonetti OP. Cardiovascular magnetic resonance findings in competitive athletes recovering from COVID-19 infection. JAMA Cardiol. (2020). 10.1001/jamacardio.2020.4916 32915194 PMC7489396

[8] Vago H Szabo L Dohy Z Merkely B. Cardiac magnetic resonance findings in patients recovered from COVID-19: initial experiences in elite athletes. JACC Cardiovasc Imaging. (2020) 14:1279–81. 10.1016/j.jcmg.2020.11.014 33341416 PMC7837171

[9] Martinez MW Tucker AM Bloom OJ Green G Difiori JP Solomon G. Prevalence of inflammatory heart disease among professional athletes with prior COVID-19 infection who received systematic return-to-play cardiac screening. JAMA Cardiol. (2021) 6:745–52. 10.1001/jamacardio.2021.0565 33662103 PMC7934073

[10] Moulson N Petek BJ Drezner JA Harmon KG Kliethermes SA Patel MR. SARS-CoV-2 cardiac involvement in young competitive athletes. Circulation. (2021) 144:256–66. 10.1161/CIRCULATIONAHA.121.054824 33866822 PMC8300154

[11] Brito D Meester S Yanamala N Patel HB Balcik BJ Casaclang-Verzosa G. High prevalence of pericardial involvement in college student athletes recovering from COVID-19. JACC Cardiovasc Imaging. (2020) 14:541–55. 10.1016/j.jcmg.2020.10.023 33223496 PMC7641597

[12] NIH. Coronavirus Disease 2019 (COVID-19). Treatment Guidelines (2019:130). Avaliable online at: https://covid19treatmentguidelines.nih.gov/ (accessed May 12, 2021).

[13] Mosteller RD. Simplified calculation of body-surface area. N Engl J Med. (1987) 317:1098. 10.1056/NEJM198710223171717 3657876

[14] Gu XS Rosenbaum PR. Comparison of multivariate matching methods: structures, distances, and algorithms. J Comput Graph Stat. (1993) 2:405–20. 10.1080/10618600.1993.10474623

[15] Lang RM Badano LP Mor-Avi V Afilalo J Armstrong A Ernande L. Recommendations for cardiac chamber quantification by echocardiography in adults: an update from the American society of echocardiography and the European association of cardiovascular imaging. J Am Soc Echocardiogr. (2015) 28:1–39. 10.1016/j.echo.2014.10.003 25559473

[16] Rudski LG Lai WW Afilalo J Hua L Handschumacher MD Chandrasekaran K. Guidelines for the echocardiographic assessment of the right heart in adults: a report from the American society of echocardiography endorsed by the European association of echocardiography, a registered branch of the European society of cardiology, and the canadian society of echocardiography. J Am Soc Echocardiogr. (2010) 23:685–713. 10.1016/j.echo.2010.05.010 20620859

[17] Boscolo-Rizzo P Borsetto D Fabbris C Spinato G Frezza D Menegaldo A. Evolution of altered sense of smell or taste in patients with mildly symptomatic COVID-19. JAMA Otolaryngol Head Neck Surg. (2020) 146:729–32. 10.1001/jamaoto.2020.1379 32614442 PMC7333173

[18] Shi S Qin M Shen B Cai Y Liu T Yang F. Association of cardiac injury with mortality in hospitalized patients with COVID-19 in Wuhan, China. JAMA Cardiol. (2020) 5:802–10. 10.1001/jamacardio.2020.0950 32211816 PMC7097841

[19] Parohan M Yaghoubi S Seraji A. Cardiac injury is associated with severe outcome and death in patients with coronavirus disease 2019 (COVID-19) infection: a systematic review and meta-analysis of observational studies. Eur Heart J Acute Cardiovasc Care. (2020) 9:665–77. 10.1177/2048872620937165 32567326 PMC7678334

[20] Puntmann VO Carerj ML Wieters I Fahim M Arendt C Hoffmann J. Outcomes of cardiovascular magnetic resonance imaging in patients recently recovered from coronavirus disease 2019 (COVID-19). JAMA Cardiol. (2020) 5:1265–73. 10.1001/jamacardio.2020.3557 32730619 PMC7385689

[21] Maron BJ Pelliccia A Spataro A Granata M. Reduction in left ventricular wall thickness after deconditioning in highly trained olympic athletes. Br Heart J. (1993) 69:125–8. 10.1136/hrt.69.2.125 8435237 PMC1024938

[22] Olah A Kovacs A Lux A Tokodi M Braun S Lakatos BK. Characterization of the dynamic changes in left ventricular morphology and function induced by exercise training and detraining. Int J Cardiol. (2019) 277:178–85. 10.1016/j.ijcard.2018.10.092 30442376

[23] Lakatos BK Molnár AÁ Kiss O Sydó N Tokodi M Solymossi B. Relationship between cardiac remodeling and exercise capacity in elite athletes: incremental value of left atrial morphology and function assessed by three-dimensional echocardiography. J Am Soc Echocardiogr. (2020) 33:101–9. 10.1016/j.echo.2019.07.017 31585830

[24] Ackermann M Verleden SE Kuehnel M Haverich A Welte T Laenger F. Pulmonary vascular endothelialitis, thrombosis, and angiogenesis in Covid-19. N Engl J Med. (2020) 383:120–8. 10.1056/NEJMoa2015432 32437596 PMC7412750

[25] Sato K Ayache A Kumar A Cremer PC Griffin B Popovic ZB. Improvement in left ventricular mechanics following medical treatment of constrictive pericarditis. Heart. (2021) 107:828–35. 10.1136/heartjnl-2020-317304 33408090

[26] Syed FF Schaff HV Oh JK. Constrictive pericarditis–a curable diastolic heart failure. Nat Rev Cardiol. (2014) 11:530–44. 10.1038/nrcardio.2014.100 25072910

[27] Kusunose K Dahiya A Popovic ZB Motoki H Alraies MC Zurick AO. Biventricular mechanics in constrictive pericarditis comparison with restrictive cardiomyopathy and impact of pericardiectomy. Circ Cardiovasc Imaging. (2013) 6:399–406. 10.1161/CIRCIMAGING.112.000078 23532508

[28] Argulian EDH. “Hot septum” sign of constrictive pericarditis. JACC Case Reports. (2020) 2:186–90. 10.1016/j.jaccas.2019.12.024 34317202 PMC8298570

[29] Matsuyama K Matsumoto M Sugita T Nishizawa J Yoshioka T Tokuda Y. Clinical characteristics of patients with constrictive pericarditis after coronary bypass surgery. Jpn Circ J. (2001) 65:480–2. 10.1253/jcj.65.480 11407725

[30] Wilson MG Hull JH Rogers J Pollock N Dodd M Haines J. Cardiorespiratory considerations for return-to-play in elite athletes after COVID-19 infection: a practical guide for sport and exercise medicine physicians. Br J Sports Med. (2020) 54:1157–61. 10.1136/bjsports-2020-102710 32878870 PMC7513247

