# Supplementary material for: Frequent Constriction-Like Echocardiographic Findings in Elite Athletes Following Mild COVID-19: A Propensity Score-Matched Analysis
Source: Front Cardiovasc Med. 2022 Jan 5;8:760651. doi: 10.3389/fcvm.2021.760651 (PMC8767617; doi:10.3389/fcvm.2021.760651)
Supplement: Supplementary file 1 [file Table_1.docx]

**Supplementary Table 1: main symptoms during COVID-19 infection in the post-COVID athlete group**

|  | Number of athletes (% of total) |
| --- | --- |
| Symptomatic | 48 (45%) |
| Febrile or subfebrile | 35 (33%) |
| Coughing | 8 (7%) |
| Loss of smell and taste | 55 (51%) |
| Chest dyscomfort | 11 (10%) |
| Dyspnea | 7 (7%) |
| Fatigue | 31 (29%) |
| Headache | 42 (39%) |
