# Supplementary material for: Frequent Constriction-Like Echocardiographic Findings in Elite Athletes Following Mild COVID-19: A Propensity Score-Matched Analysis
Source: Front Cardiovasc Med. 2022 Jan 5;8:760651. doi: 10.3389/fcvm.2021.760651 (PMC8767617; doi:10.3389/fcvm.2021.760651)
Supplement: Supplementary file 2 [file Table_2.docx]

**Supplementary Table 2: Baseline and conventional echocardiographic characteristics of post-COVID athletes with versus without septal flattening**

|  | Post-COVID with SF (n=35) | Post-COVID without SF (n=72) | p value |
| --- | --- | --- | --- |
| Age (years) | 20.9±3.9 | 23.8±6.7 | **<0.05** |
| Female (n [%]) | 7 (21%) | 19 (26%) | 0.54 |
| Height (cm) | 183.4±9.0 | 182.7±10.5 | 0.74 |
| Weight (kg) | 77.7±11.2 | 81.5±16.9 | 0.23 |
| BSA (m²) | 1.99±0.18 | 2.02±0.27 | 0.41 |
| SBP (mmHg) | 132.1±14.2 | 129.3±15.7 | 0.39 |
| DBP (mmHg) | 78.1±10.9 | 80.2±11.4 | 0.38 |
| HR (1/min) | 62.0±9.6 | 63.4±11.1 | 0.54 |
| Training per week (hours) | 12.8±5.9 | 13.2±6.0 | 0.79 |
| LVIDd (mm) | 51.9±4.6 | 51.8±4.4 | 0.89 |
| IVSd (mm) | 9.1±1.6 | 9.6±1.9 | 0.15 |
| PWd (mm) | 8.1±1.2 | 8.5±1.4 | 0.17 |
| RWT (%) | 0.32±0.05 | 0.33±0.05 | 0.15 |
| LAVi (mL/m²) | 26.5±7.4 | 26.4±6.1 | 0.90 |
| Transmitral E wave (cm/s) | 85.6±17.6 | 79.6±14.8 | 0.05 |
| Transmitral A wave (cm/s) | 48.5±12.0 | 51.0±12.4 | 0.31 |
| E/A | 1.83±0.41 | 1.62±0.40 | **<0.05** |
| DT (ms) | 185.1±35.7 | 196.5±42.8 | 0.18 |
| E/e’ average | 4.7±0.9 | 4.6±0.9 | 0.91 |
| Mitral lateral s’ (cm/s) | 13.0±2.5 | 12.6±2.4 | 0.42 |
| Mitral lateral e’ (cm/s) | 20.8±2.9 | 19.3±3.4 | **<0.05** |
| Mitral lateral a’ (cm/s) | 7.6±1.8 | 8.6±2.0 | **<0.05** |
| Mitral medial s’ (cm/s) | 13.0±2.4 | 12.6±2.5 | 0.42 |
| Mitral medial e’ (cm/s) | 16.3±2.5 | 15.3±2.8 | 0.08 |
| Mitral medial a’ (cm/s) | 8.0±1.3 | 8.6±1.4 | 0.05 |
| e’ lateral/e’ septal | 1.29±0.22 | 1.30±0.20 | 0.67 |
| RV basal diameter (mm) | 33.9±4.5 | 34.5±4.1 | 0.44 |
| TAPSE (mm) | 24.4±4.2 | 24.9±3.8 | 0.50 |
| RAVi (mL/m²) | 29.7±7.8 | 27.2±5.8 | 0.06 |
| PASP (mmHg) | 21.9±4.3 | 20.2±4.3 | 0.10 |
| PADP (mmHg) | 7.8±2.8 | 6.4±1.8 | **<0.05** |
| PAMP (mmHg) | 15.1±3.8 | 12.7±4.2 | 0.05 |
| IVC max (mm) | 13.3±3.1 | 13.0±3.0 | 0.72 |
| IVC min (mm) | 11.6±5.4 | 10.7±8.7 | 0.84 |
| RAP (mmHg) | 4.1±2.1 | 3.2±1.5 | **<0.05** |
| RVOT VTI (cm) | 20.3±3.2 | 19.8±3.7 | 0.53 |
| PVR (Wood units) | 1.21±0.20 | 1.25±0.21 | 0.50 |
| TAPSE/PASP | 1.15±0.25 | 1.26±0.31 | 0.11 |
| Prevalence of mild pericardial effusion (n [%]) | 14 (41%) | 27 (37%) | 0.80 |

*Abbreviations*: SF = septal flattening; BSA = body surface area; SBP = systolic blood pressure; DBP = diastolic blood pressure. HR = heart rate; LVIDd = left ventricular end-diastolic diameter; IVSd = interventricular septal thickness; PWd = posterior wall thickness; RWT = relative wall thickness; LAVi = left atrial volume index; DT: deceleration time; LV eccentricity index = left ventricular eccentricity index; RV basal diamater = right ventricular basal diameter; TAPSE = tricuspid annular plane systolic excursion; RAVi = right atrial volume index; PASP = pulmonary arterial systolic pressure; PADP = pulmonary arterial diastolic pressure; PAMP = pulmonary arterial mean pressure; IVC = inferior vena cava; RAP = right atrial pressure; RVOT VTI = right ventricular outflow tract velocity-time integral; PVR = pulmonary vascular resistance
