# Supplementary material for: Frequent Constriction-Like Echocardiographic Findings in Elite Athletes Following Mild COVID-19: A Propensity Score-Matched Analysis
Source: Front Cardiovasc Med. 2022 Jan 5;8:760651. doi: 10.3389/fcvm.2021.760651 (PMC8767617; doi:10.3389/fcvm.2021.760651)
Supplement: Supplementary file 3 [file Table_3.docx]

**Supplementary Table 3: Case presentations of 5 post-COVID athletes with septal flattening: echocardiographic and cardiac magnetic resonance imaging findings (figures and videos are available at figshare.com by clicking on the case number)**

| Athlete number | Sex | Age | Sport discipline | Past medical history | Symptoms during infection | Echocardiography findings | cMR findings |
| --- | --- | --- | --- | --- | --- | --- | --- |
| [#1](https://figshare.com/s/bc1babcc84afadf8ad16) | male | 20 | water polo | none | none | 3D LVEF: 52.0%  EI: 1.22  FW/S LS: 0.94 | No apparent abnormality^1^ |
| [#2](https://figshare.com/s/a299d683012a7d91794c) | female | 22 | handball | none | headache | 3D LVEF: 55.1%  EI: 1.27  FW/S LS: 0.90 | No apparent abnormality^1^ |
| [#3](https://figshare.com/s/091b3211603d9de3a60e) | female | 24 | handball | none | loss of taste and smell | 3D LV EF: 61.8%  EI: 1.32  FW/S LS: 0.87 | No apparent abnormality^1^ |
| [#4](https://figshare.com/s/418ff25932af2bbf9683) | female | 26 | handball | none | fever for 12 hours | 3D LV EF: 64.6%  EI: 1.39  FW/S LS: 0.84 | No apparent abnormality^1^ |
| [#5](https://figshare.com/s/d02b4404e6ae3f2d7a95) | male | 17 | water polo | none | none | 3D LV EF: 57.4%  EI: 1.27  FW/S LS: 0.88 | No apparent abnormality^1^ |

^1^: No signs of any typical features of pericardial inflammation, including thickened pericardium, significant pericardial effusion, increased signal intensity showing edema on T2 weighted images, or scarring/necrosis apparent on late gadolinium enhanced images

*Abbreviations*: cMR = cardiac magnetic resonance imaging, LVEF = left ventricular ejection fraction, EI = eccentricity index, FW/S LS = free wall-to-septal longitudinal strain ratio
