# Supplementary material for: Frequent Constriction-Like Echocardiographic Findings in Elite Athletes Following Mild COVID-19: A Propensity Score-Matched Analysis
Source: Front Cardiovasc Med. 2022 Jan 5;8:760651. doi: 10.3389/fcvm.2021.760651 (PMC8767617; doi:10.3389/fcvm.2021.760651)
Supplement: Supplementary file 4 [file Table_4.docx]

**Supplementary Table 4: Intra- and interobserver variability of the key parameters**

|  | Intraobserver variability | | Interobserver variability | |
| --- | --- | --- | --- | --- |
|  | **ICC** | **CV** | **ICC** | **CV** |
| 3D LVEDV | 0.959 | 1.9% | 0.922 | 8.08% |
| 3D LVESV | 0.869 | 2.8% | 0.755 | 17.2% |
| 2D LVGLS | 0.896 | 3.6% | 0.892 | 3.6% |

*Abbreviations*: ICC = intraclass correlation coefficient; CV = coefficient of variation; LVEDVi = left ventricular end-diastolic volume; LVESVi = left ventricular end-systolic volume; LVGLS = left ventricular global longitudinal strain
